# Amelogenesis imperfecta: therapeutic strategy from primary to permanent dentition across case reports

**DOI:** 10.1186/s12903-018-0554-y

**Published:** 2018-06-15

**Authors:** Steve Toupenay, Benjamin Philippe Fournier, Marie-Cécile Manière, Chantal Ifi-Naulin, Ariane Berdal, Muriel de La Dure– Molla

**Affiliations:** 1 Centre de référence des maladies rares orales et dentaires Orares, Hopital Rothschild, APHP, Paris, France; 2 UFR d’Odontologie, Université Paris-Diderot, F-75006 Paris, France; 3 Université Paris-Descartes, F-75006 Paris, France; 4 Université Pierre et Marie Curie-Paris, F-75006 Paris, France; 5 Centre de Recherche des Cordeliers, INSERM UMRS 1138, Laboratory of Molecular Oral Pathophysiology, F-75006 Paris, France; 6 INSERM UMR_S1163 Bases moléculaires et physiopathologiques des ostéochondrodysplasies, Institut Imagine, Necker, Paris, France; 7 Hôpitaux Universitaires de Strasbourg, Pôle de Médecine et Chirurgie Bucco-Dentaires, Centre de Référence des Maladies Rares Orales et Dentaires, CRMR O-Rares, Strasbourg, France; 8 Faculté de Chirurgie Dentaire, Université de Strasbourg, Strasbourg, France; 9 Odontology Department, Rothschild Hospital, 5 rue Santerre, 75012 Paris, France

**Keywords:** Amelogenesis imperfecta, Dental care, Operative dentistry, Paediatric dentistry

## Abstract

**Background:**

Hereditary enamel defect diseases are regrouped under the name “Amelogenesis Imperfecta” (AIH). Both dentitions are affected. Clinical expression is heterogeneous and varies between patients. Mutations responsible for this multigene disease may alter various genes and the inheritance can be either autosomal dominant or recessive, or X-linked. Until now, no therapeutic consensus has emerged for this rare disease.

**Case presentation:**

The purpose of this article was to report treatments of AIH patients from childhood to early adulthood. Treatment of three patients of 3, 8 16 years old are described. Each therapeutic option was discussed according to patients’ age and type of enamel alteration. Paediatric crowns and resin based bonding must be preferred in primary teeth. In permanent teeth, non-invasive or minimally invasive dentistry should be the first choice in order to follow a therapeutic gradient from the less invasive options to prosthodontic treatments.

**Conclusion:**

Functional and aesthetic issues require patients to be treated; this clinical care should be provided as early as possible to enable a harmonious growth of the maxillofacial complex and to prevent pain.

## Background

Amelogenesis imperfecta is a rare genetic disease affecting enamel. Primary and permanent teeth are concerned with almost the same severity. Differential diagnosis must be made with enamel developmental defects caused by environmental factors (fluoride, tetracycline???) [1] or traumatic etiologies as they will only affect defined teeth and rarely both dentitions. For example, experimental studies showed that molar incisor hypoplasia (MIH), which only affects permanent incisors and first molars, might be caused by prenatal or early child exposure to endocrine disruptors [2].

Amelogenesis imperfecta presents large variability in its clinical expression. Mutations have been reported in different genes. Some of them encode for enamel proteins, either structural (amelogenin, enamelin, ameloblastin, c4orf26) or enzymatic (kallikrein 4*, MMP20*); some others encode for transcription factors (*MSX2, DLX3*), cellular proteins (*WDR72, FAM83H, COL17A1*), cellular receptor (*ITGB6*) and calcium carrier (*SLC24A4*) [3]. Until today, no relation between genotype and phenotype has been established. Enamel may be modified in its width, microstructure or mineralization degree. Thus, clinical symptomatology goes from light discoloration to disintegration/breakdown of the enamel of the entire tooth. Witkop’s classification distinguished 4 different types: hypoplastic, hypomature, hypomineralized and hypomature with taurodontism forms, with 14 specific subtypes [4]. Indeed we differentiate 3 clinical entities: hypoplastic, hypomature and hypomineralized AI.

- Hypoplastic AIH (type I) consists of quantitative alteration of enamel with localized or generalized reduced thickness. Teeth are yellow to light brown, surface is rough with pits or larger area defects. Severe hypoplastic phenotype leads to morphological anomalies seen on radiographic examinations. No pain is associated with this AI, although some slight thermal sensitivity may sometimes be reported [5].
- Hypomature AIH (type II) consists of a defect in matrix protein degradation. In enamel, which is the most calcified structure in the organism, proteins must be degraded and removed to achieve final crystal growth. In type II, enamel appears white or brown, without translucency. Hardness during probing and thickness of enamel layer are normal. However, enamel breakdown often occurs. On radiographs, enamel opacity is decreased especially near the enamel dentin junction. This type of AIH is the mildest form and frequently undiagnosed. Aesthetics is the first cause of consultation [6].
- Hypomineralized AIH (type III) is the most severe AI form. Enamel mineral content is reduced causing pain while masticating, and brushing. Gingivitis and periodontal diseases have been described, with large amounts of dental calculus. Teeth are very sensitive to temperature and brushing. Enamel is dark yellow or brown. On radiographs, enamel and dentin may reach the same radiodensity [7]. Anxiety has often been reported in these patients due to permanent dental pain [8].

Other dental anomalies may be associated with AI [9]: taurodontism [10], pulp stones, delayed tooth eruption, anterior open bite or craniofacial anomaly [11, 12]. Surprisingly, no increased incidence of caries has been reported.

## Case presentation

### Case report 1

**Fig. 1:**
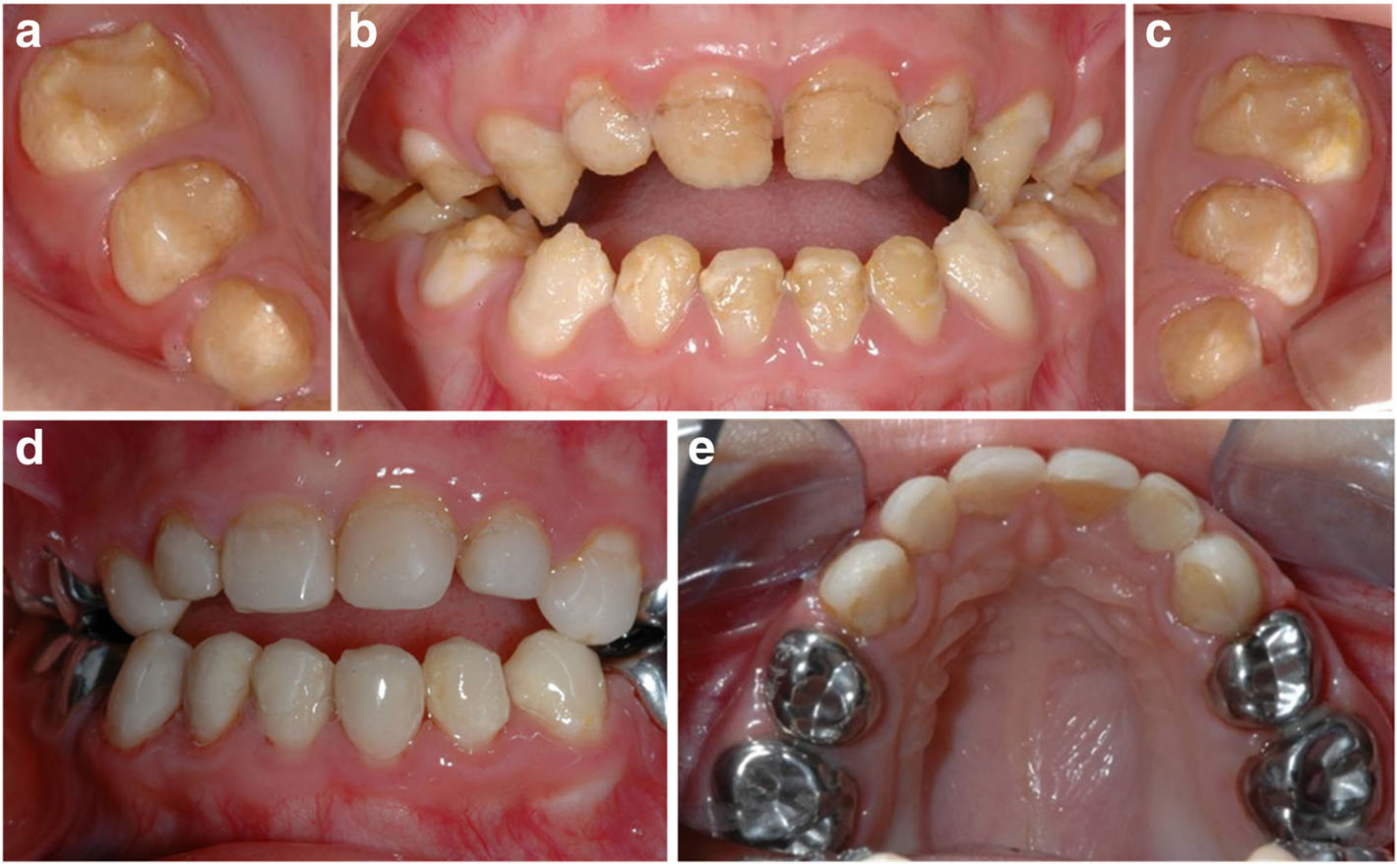
4,5-year-old patient affected by hypomineralized AI. Clinical examination revealed pain during brushing and hot and cold sensitivity, open bite whithout digit sucking. **a**–**c** Enamel was yellow to brown, easily chipping, with loss of dental morphology. **d**, **e** Oral surgery was realized under local anesthesia through four visits. Stainless steel pediatric crowns were realized on primary molars, and direct composite restorations were done in anterior teeth

A three-year-old girl was referred to the Reference Centre of Rare Diseases in Paris. Her medical history was noncontributory. According to her mother, she complained with pain while eating, moderate sensitivity during tooth brushing and above all poor aesthetic aspect of her teeth. Intraoral examination revealed a hypoplastic AIH with yellow teeth and rough surfaces (Fig. 1a). Brown extrinsic discoloration was seen in the hypoplastic area. Enamel was reduced in thickness and severely hypoplastic, giving the idea of a false microdontia with multiple diastemas. Molars were the most affected teeth showing reduced crown height. In addition, anterior open bite was noted without thumb sucking. Treatment was planned following 3 objectives at this age:

- Pain prevention and treatment
- Protection of dental tissue integrity in order to maintain occlusal function and limit dental biofilm retention
- Restoration of smile aesthetics.

On primary molars, the choice of treatment was stainless steel crowns (3 M™ ESPE™) because the occlusal morphology was lost (Fig. 1b). This way, vertical dimension was slightly increased and maintained. The incisors and canines were isolated with a rubber dam and direct dental composite restorations were placed (Herculite, Kerr [13, 14] with ER2 adhesives Optibond SL). Teeth were not prepared; we etched with 35% Phosphatidic acid for 30 s, rinsed for 30 s with air and water. Then teeth were air dried, adhesive was applied with an applicator tip, excesses were removed with air before polymerization for 45 s. Affected enamel was not removed but bonding was directly applied to it. As enamel surface appeared rough, a flow composite (Tetric Evoflow, Ivoclar) was applied and served as intermediate material. Its higher fluidity and wettability would allow penetrating enamel roughness (Fig. 1b). Because tooth morphology of anterior teeth was not severely altered, “Odus” molds were not useful to offer a correct restoration. Composite resins were applied in one layer. Finishing and polishing were achieved with abrasive discs (Sof-lex/3 M ESPE). Patient follow-ups were done 6 months and 1 year after treatment. Composite sealing and oral hygiene were controlled.

### Case report 2

**Fig. 2:**
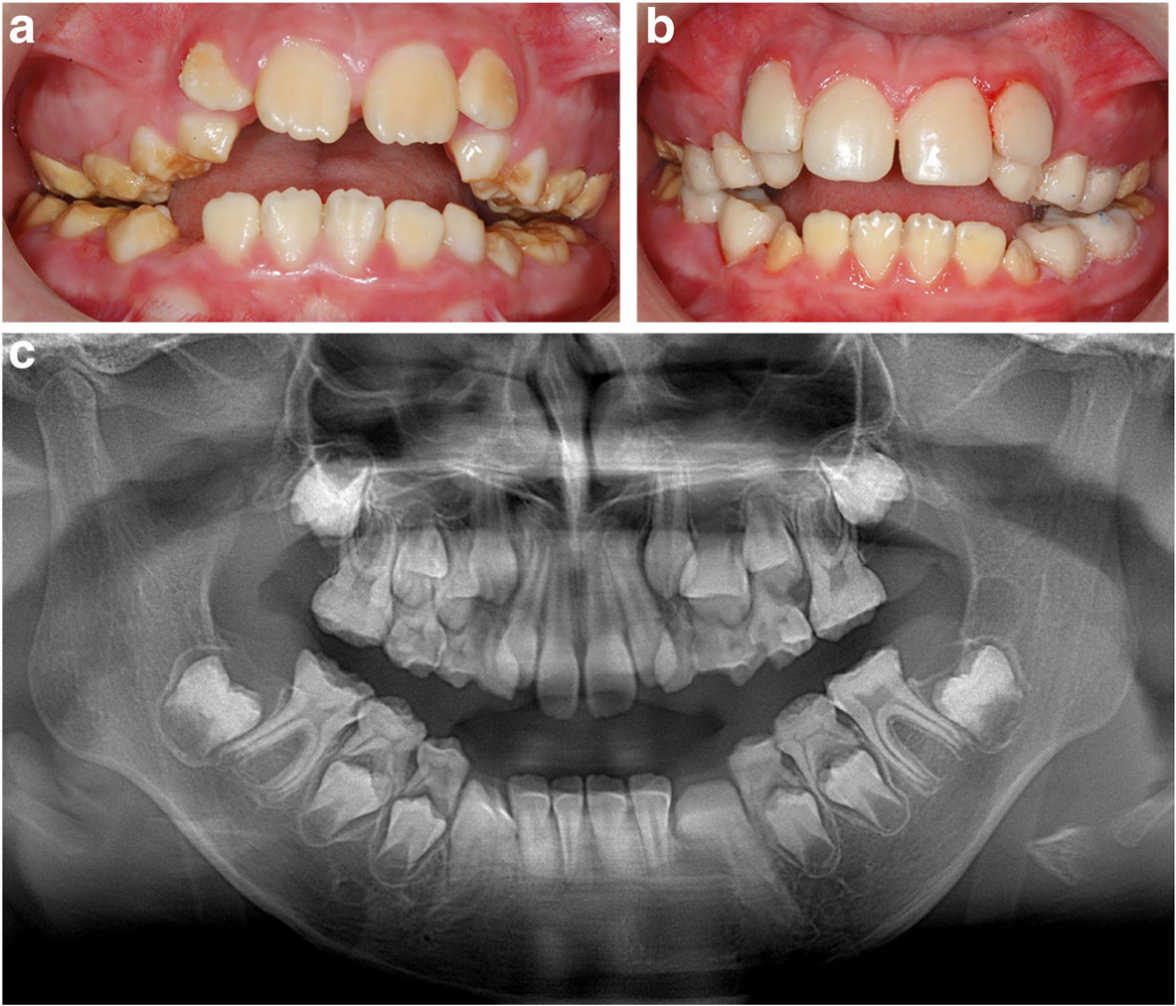
8-year-old patient with hypomineralized AI. **a** Oral examination revealed brown enamel with severe breakdown in primary teeth. Patient history shows pain while eating, brushing and also breathing. Aesthetic complaint was high because of laughing at school. **b** Composite veneers and complete composite crowns were realised on anterior permanent teeth and posterior primary teeth respectively. **c** panoramic radiograph revealed severe reduction of enamel layer

An 8-year-old patient referred to the Reference Centre of Rare Diseases, Paris. Her medical and familial history revealed no etiologic explanation. Her main complaint was extreme sensitivity to hot and cold and she was anxious about dental care for this reason. Oral clinical exam showed a mixed dentition, with eruption of permanent incisors and first molars. Hypomineralized AI was diagnosed (Fig. 2a). Enamel was dark yellow in permanent teeth and brown in primary teeth. Some enamel breaks were observed in posterior teeth. A severe open bite was observed, associated with only occlusal contacts on first permanent molars and second primary molars. Maxillary bone showed insufficient transversal growth. Facial and oral functional exams revealed buccal breathing and nocturnal snoring explaining the ectopic maxillary lateral incisor eruption in the vestibular area. The patient was referred to the otorhinolaryngology department to investigate obstructive sleep apnea syndrome. The panoramic radiograph showed a reduction in the enamel thickness as well as a similar X-ray density between hypomineralized AI and dentin (Fig.2c). The patient showed very low self-esteem because of her poor appearance. She reported bullying at school and didn’t want to smile.

Multidisciplinary treatment objectives taken into account at this age were:

- Preservation of tooth integrity and vitality of permanent teeth emerged in the oral cavity
- Non-invasive rehabilitation that allowed evolution during growth
- Restoration of smile aesthetics
- Normalization of oral function (mastication, respiration, swallowing)

Because of the strong aesthetic request, full composite rehabilitation was decided (Fig. 2b). Master impression of the two arches was recorded with silicone material. Hard plaster (Type IV) was used, models were adjusted to a semi-adjustable articulator using a centric relation record. Rehabilitation of anterior teeth was done first in order to obtain the patient’s confidence. This was workable because of the absence of anterior occlusion. Indirect resin-based composite (Premise Indirect System, Kerr) facets were performed on maxillary incisors without tooth cavity preparation. A layer of an opaque shade of composite was applied to mask the remaining spot. Composite resin A3 shade was used cervically, A2 in the core and A1 in the incisal edge. Careful polishing was made especially at the gingival border with a Touati bur. In primary teeth, full composite crowns were still build-up in plaster models. The restoration was bonded using dual cured composite resin (Variolink Esthetic, Ivoclar™ Vivadent™). Occlusion was lightly increased to create enough space for this restorative reconstruction. Stainless steel crowns (3 M™ ESPE™) were applied to all first permanent molars without tooth preparation and sealed with glass ionomer cement. Orthopedic treatment followed to treat the maxillary hypoplasia.

### Case report 3

**Fig. 3:**
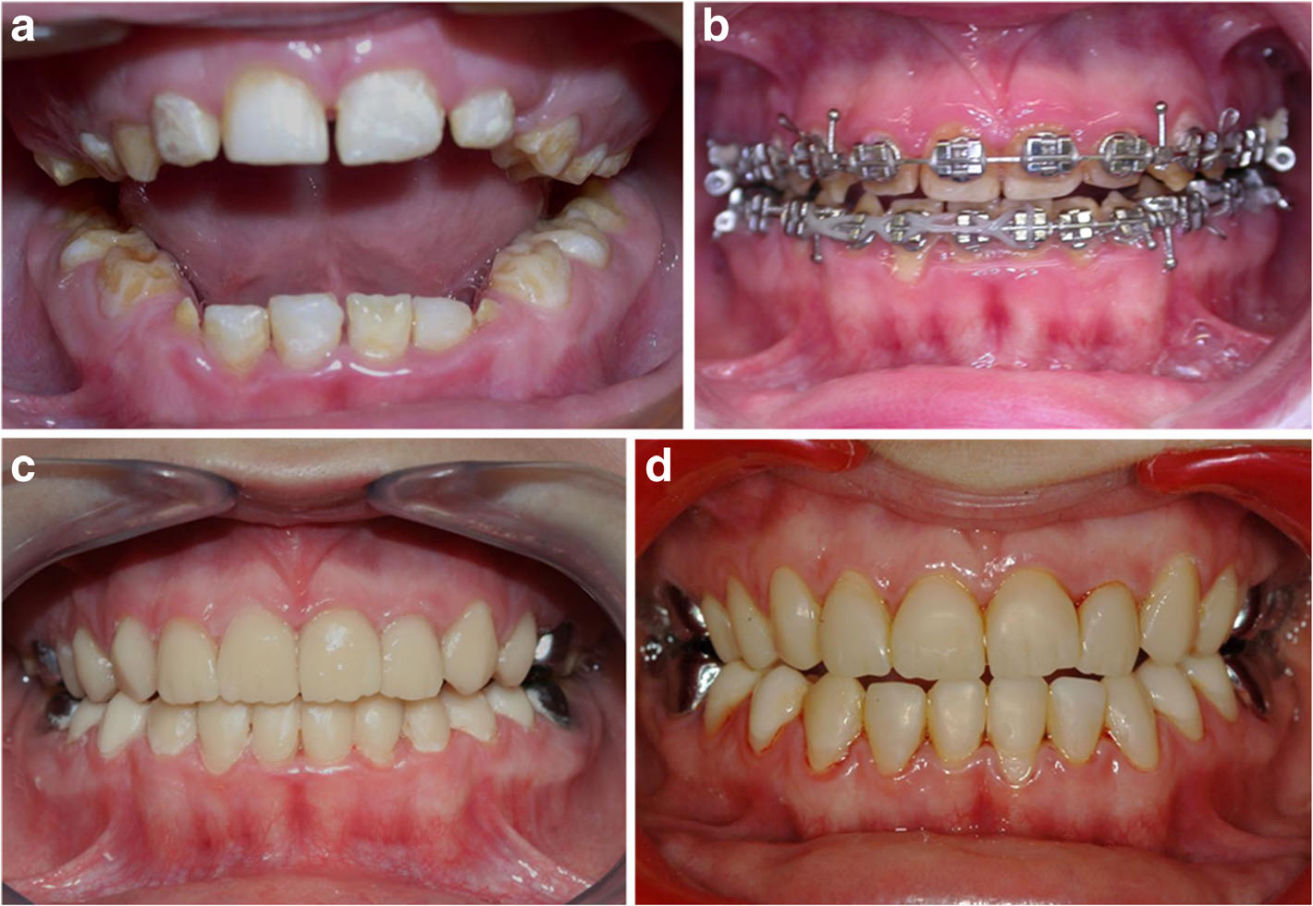
Hypoplastic amelogenesis imperfecta associated to open bite patient (**a**): 9 years old was treated by an orthodontic treatment at 13 years old (**b**). At the end of the treatment, indirect composite restorations were realized with veneers on anterior teeth and full composite crowns on premolars (**c**: 16 years old). Stainless steel crowns had been previously realized on the first permanent molars at the age of 7. View of the patient 5 years later (**d**)

A 16-year-old girl was referred by an orthodontist to the Reference Centre of Rare Diseases in Paris. Orthodontic treatment was performed with classical bracket technique in order to close anterior open bite (Fig. 3a-b). At the end of the treatment, the patient requested full mouth rehabilitation. She complained first of all about aesthetics but she also reported difficulties and painful chewing. Intraoral examination revealed hypomineralized AI associated with some hypoplasia. A little open bite remained after orthodontic treatment. Teeth were small with diastemas that were not closed as requested by the practitioner. In this occlusal context dental rehabilitation may be done without teeth reduction. Treatment was discussed according to several objectives taking into account the patient’s age:

- Functional restoration
- Aesthetic restoration
- Lasting treatment
- Minimally invasive treatment

Master impression of the two arches was recorded with a silicone material and working cast was mounted onto a semi-adjustable articulator using a centric relation record. Composite veneers were applied on incisors and composite full crowns on all other teeth (Fig. 3c). Nanohybrid indirect composite (Premise Indirect System, Kerr) was used with dentin and enamel shades mimicking the clinical shade (A3 shade was used cervically, A2 in the core and A1 in the incisal edge). Each layer was polymerised. Rigorous polishing was done in order to obtain shiny surfaces (Tool kit, Kulzer). The restoration was bonded using dual cured composite resin (Variolink Esthetic, Ivoclar™ Vivadent™) taking care to separate each proximal contact with metal matrix. Carefully polishing was made especially at the gingival border with a Touati bur. The patient was very satisfied with the aesthetic appearance. She did not report any trouble with mastication. She was followed every 6 months. Oral hygiene and integrity of the restoration were scrupulously monitored. Direct composite was applied 3 years later, on the cervical part of the crown because gingival maturation occurred. She had only difficulty to control calculus deposition on the lingual part of mandibular incisors. Five years later, the restorations were still satisfactory (Fig. 3d).

## Discussion and conclusion

Guidelines for AI treatment have been established by AAPD (American Academy of Pediatric Dentistry) [15]. Factors such as age, socio-economic conditions, AI type and severity have to be taken into account in treatment planning. Patients’ first appointment usually corresponded to establishment determining the age of primary, mixed and permanent dentitions (that is 4, 8 and 13 year-old, respectively), and the two main demands were pain and aesthetics [16]. These patients suffered from reduced quality of life, social integration difficulties and loss of self-esteem [17]. Oral hygiene and rigorous follow-up are recommended. Hypomineralized enamel showed progress alteration with time because of its softness. Composite fillings can limit this degradation. Dental rehabilitation is still important to improve oral health in children. Rough enamel is associated with dental plaque retention, increasing gingival inflammation and pain. Hypomineralized enamel is the most severe form: once occlusion is established, teeth wear quickly inducing large tissue losses. Patients describe eating difficulties and pain when temperature changes. Thus, efficient tooth brushing cannot be achieved / tooth brushing cannot be effective. By contrast, hypoplastic AIs mainly present unsightly teeth complaints, while in hypomineralized type, local anesthesia is required for dental scaling.

Treatment should begin as soon as possible according to patient compliance in office dental care. For very young patients, general anesthesia may be necessary. Stainless steel crowns were indicated in primary teeth with hypoplastic or hypomineralized AI in order to reduce tooth sensitivity and restore enamel loss. Composite restorations were indicated for all primary teeth. Previous studies regarding bonding to AI enamel were contradictory and varied with AI types [18, 19]. Some authors suggest complete enamel etching with sodium hypochlorite rinsing (5% during 1 min) in order to remove residual enamel proteins, especially in hypomature forms [20–22]. In vitro studies showed a decrease in bonding strength [23] while some others observed similar rupture strength values to healthy enamel ones. This latter may be explained by an increase of bonding area due to the microporosity of the affected enamel. Bonding on dentin is also different. Indeed, dentin in AI patients is more mineralized than usual, looking like reactional dentin with obliterated tubuli [24].

In mixed dentition, rehabilitation must be done as soon as teeth erupt. Treatment main goals should be the preservation of tooth integrity and vitality [25]. Paediatric crowns can be easily performed on first molars without tooth preparation, especially indicated when teeth are painful or hypoplastic. Orthodontic elastic spacer was used to separate teeth. In other cases, only prophylactic care may be enough. In hypomineralized forms, glass ionomer cements on occlusal surfaces were efficient in preventing pain and allowing temporizing until teeth eruption was achieved. Clinical follow ups should be planned every 6 months if new teeth erupt and every 9–12 months in stable periods. Orthodontic treatment is not contraindicated in AI patients. Brackets’ bonding can be made with glass ionomer cements. Open bite prevalence is increased in AI patients. Treatment is often long and might need orthognatic surgery. In mild AI forms (without any pain or important hypoplasia), definitive rehabilitation should be planned only at the end of the orthodontic treatment. In other cases, primary restoration could be done before orthodontic treatment and reassessed at the end of the treatment.

In permanent dentition, different treatments from restorative to prosthetic rehabilitation have been reported in the literature [26] (Table 1). Nevertheless, no consensus between several case reports has been reached. Before adhesive dentistry and full ceramic material arrival, prosthetic treatment with ceramic crowns was done on all teeth. This kind of treatment is no longer recommended today for young adult. Most aesthetic results were obtained with fixed prosthodontics and all ceramic restorations showed good success rates [27]. However, teeth, especially anterior teeth, have to be devitalized, which decreases their longevity. Veneers were also done on anterior teeth in order to preserve dental tissues [28–32]. Their major disadvantage is their cost and the fact that their placement is time consuming [30].

**Table 1:** Advantages and disadvantages of the therapeutic alternatives in AI dental treatment

	Advantages	Inconveniences	References
Fixed Prosthodontics	Aesthetics Occlusion Mechanical properties	Invasive Long treatment Tooth vitality Cost	Robinson et al., 2006 [32] Gisler et al., 2010 [30] Chan et al., 2011 [28] Ramos et al., 2011 [31]
Removable Prosthodontics	Fast Occlusion Cost effective	Transitory Hygiene Retention issues	Zarati et al., 2009 [33]
Resin Based Composites - Direct Restoration	Correct aesthetics Non invasive Cost effective	Mechanical properties Longevity? Occlusion regulation	Sockalingam S, 2011 [44]
Resin Based Composites-Indirect Restoration	Minimally Invasive Aesthetics (stratification, opacity) Mechanical properties Easy to repair Bite set up on simulator	Durability? Wear	Manhart J et al., 2000 [45] Koyuturk AE et al., 2013 [46]
Resin Based Composites-Indirect Restoration CAD-CAM	Same as above Possibility to use new polymer infiltrated ceramic network materials single office appointment	Same as above Steep Learning curve Occlusion	Fasbinder DJ, 2006 [47] Schlichting LH1 et al., 2011 [48]

Some authors proposed overdenture treatments [33]. In this case, occlusion and aesthetics were restored quickly. This kind of treatment is an option in mixed or young permanent dentition in order to wait for growth end. Still, overdentures should be transitory options since long term failures due to retention loss are frequent [34].

Direct or indirect [35–38] dental composites constitute other treatment options. These materials allow an aesthetic result with good long term outcomes and minimally invasive intervention [39]. Clinical reports showed short term follow-ups. Only two articles presented data with a longer follow-up [40]. Nevertheless in AI patients, the failure rate seemed to be increased compared to unaffected patients [41] or to the other dental abnormalities (for example: oligodontia or palatal clefts [42, 43]). This may be due to the less shear bond strength reported in AI teeth. A consensus protocol on AI enamel and dentin bonding is still to be decided.

AI is a rare inherited enamel disease, which explains the absence of evidence-based clinical recommendation and makes AI treatment challenging. Aesthetics, pain or tooth breakdown were the major patient complaints. Restorative to prosthodontic dentistry must be done in order to maintain oral function and growth preventing tooth loss and allowing oral hygiene maintenance. The first consultation must be as early as possible. Treatment alternatives deal with minimal invasive dentistry with the objective of maintaining tooth vitality as long as possible. The goal is to achieve therapeutic answer during the entire patient’s life. In this respect, establishing a good trust relationship between child and dentist is critical. Genetic and biological knowledge of AI physiopathology is also helpful in treatment plan decision.

## Abbreviations

AAPD: (American Academy of Pediatric Dentistry
AI: Amelogenesis imperfecta
